# Supplementary material for: Development of an Immunochromatography Assay to Detect Marburg Virus and Ravn Virus
Source: Viruses. 2023 Nov 29;15(12):2349. doi: 10.3390/v15122349 (PMC10747695; doi:10.3390/v15122349)
Supplement: Supplementary file 1 [file viruses-15-02349-s001.zip › viruses-2686418-supplementary.pdf]

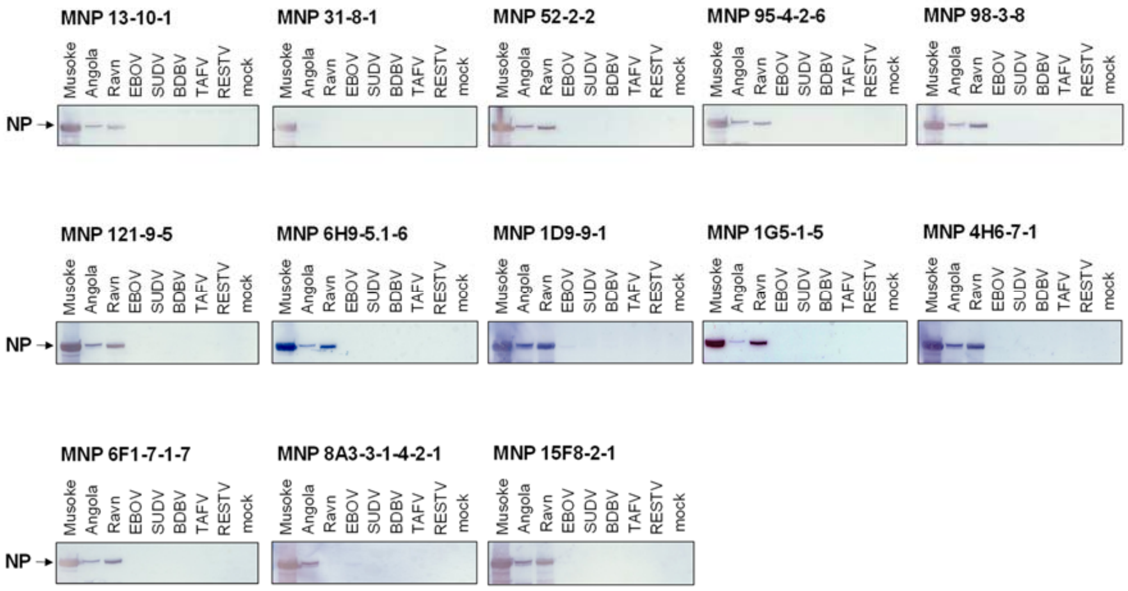

Figure S1. Binding of NP-specific mAbs in immunoblotting. rNPs of each filovirus were expressed in 293T cells and cell lysates were subjected to SDS-PAGE and Western blotting.
